# Accuracy of Orthodontic Miniscrew Implantation Assisted by Dynamic Navigation Technology Combined With Cone Beam Computed Tomography

**DOI:** 10.7759/cureus.80882

**Published:** 2025-03-20

**Authors:** Xinze Zhang, Yingying Su, Ruiqing Wu, Hao Wang, Zhihui Wang

**Affiliations:** 1 Dentistry, Beijing Tiantan Hospital, Capital Medical University, Beijing, CHN; 2 Research, Health Research Partners, Calgary, CAN; 3 Orofacial Pain, University of Rochester, Eastman Insititute for Oral Health, Rochester, USA

**Keywords:** accuracy, cbct, dynamic navigation, miniscrews, porcine

## Abstract

Objective: To evaluate the accuracy of orthodontic miniscrew placement using a dynamic navigation system combined with cone beam computed tomography (CBCT) imaging in porcine mandibles.

Methods: The Porcine mandible was scanned using CBCT. Virtual implant positions were designed using the planning software based on CBCT data. A total of 30 orthodontic miniscrews were placed in the porcine mandibles with the aid of the dynamic navigation system. A CBCT scan was taken after implantation. The deviations at entry points, endpoints. and angle deviation between the actual and planned miniscrew locations were measured by comparing the pre- and post-operation CBCT.

Results: CBCT images of post-surgical dynamic navigation guided-miniscrew placement showed no root contact (0/30). The average linear deviations at the miniscrew entry points and endpoints were 1.8 ± 0.7 mm and 1.8 ± 0.6  mm, respectively. The average angular deviation was 4.1° ± 1.6°.

Conclusion: The dynamic navigation system achieves acceptable accuracy for orthodontic miniscrew placement in porcine mandibles, reducing risks associated with implantation.

## Introduction

In the past few decades, orthodontic miniscrews have been widely used as a primary orthodontic technique due to their ability to provide absolute anchorage. However, the use of orthodontic miniscrews carries various risks, such as damage to anatomical structures, loss of stability, and failure of osseointegration [1]. The main challenges for orthodontists when using miniscrews are ensuring a high success rate and minimizing the risk of damaging adjacent roots [2].

In recent years, digital orthodontics has become increasingly popular, transforming conventional workflows. These new technologies enable orthodontists to protect patients at every step of the treatment process, from diagnosis and treatment planning to customized production and outcome evaluation [3-6]. Currently, one of the main methods to improve the accuracy of implant placement involves the use of guide plate systems [7]. However, the instability of the guide plate, the inability to adjust the implant angle intraoperatively, and the lack of direct surgical visibility have posed challenges to the accuracy and safety of miniscrew implantation [8].

With the advancement of new technologies, dynamic navigation systems have rapidly developed in the field of dental implantology [9]. An increasing number of implantologists have begun to apply this technology in clinical surgeries, but studies evaluating its accuracy in orthodontic miniscrew implantation remain scarce.

This study aims to analyze the accuracy of orthodontic miniscrew implantation using a dynamic navigation system combined with cone beam computed tomography (CBCT) imaging in porcine mandibles. The findings are expected to provide practical insights into clinical applications of this technology in orthodontics.

## Materials and methods

Experimental subjects

One porcine mandible with intact soft tissues obtained from a local butcher shop was selected as the experimental subject. According to the National Institutes of Health (NIH) guidelines, no protocol is required for tissues from deceased animals.

Experimental equipment

The experimental equipment included the following: 1) Dentsply Sirona (Charlotte, NC) oral implant navigation system (consisting of a main unit, implant handpiece locator, reference plate, navigation tool kit, and implant drill guide); 2) Dentsply Sirona implant accuracy verification software; 3) self-tapping orthodontic miniscrews (specifications: 1.4 × 8.0 mm and 2.0 × 10 mm; Ormco Corporation, Orange, CA); and 4) CBCT Dentrix 50 (DentaLink, Shenzhen, China).

Experimental methods

Design of Miniscrew Implantation With Dynamic Real-Time Navigation

The locator was first placed on the porcine mandible, and CBCT scans were taken at 85 kV, 9 mA tube current, 0.25 mm pixel size with 1.25 mm slice thickness, and field of view of 15 x 9 cm. The scanned data were imported into the navigation software, and a digital 3D model of the porcine mandible was constructed. The 3D positions of orthodontic miniscrews were designed based on the anatomical structure of the alveolar bone, the position and morphology of tooth roots, the distance between roots, and the expected direction and extent of tooth movement.

Using principles such as maintaining a distance of 5-7 mm from the alveolar crest and angling 30-40° from the long axis of the tooth, the positions and orientations of 30 miniscrews were virtually designed (implanted in three batches, 10 screws per batch). The reference plate and registration plate were installed on the porcine mandible, and the implant handpiece locator was paired with the navigation system. The orthodontic miniscrews were then implanted into the porcine mandibles. After implantation, CBCT scans were taken to verify the accuracy of the miniscrew positions.

Miniscrew Implantation With Dynamic Real-Time Navigation

**Figure 1:**
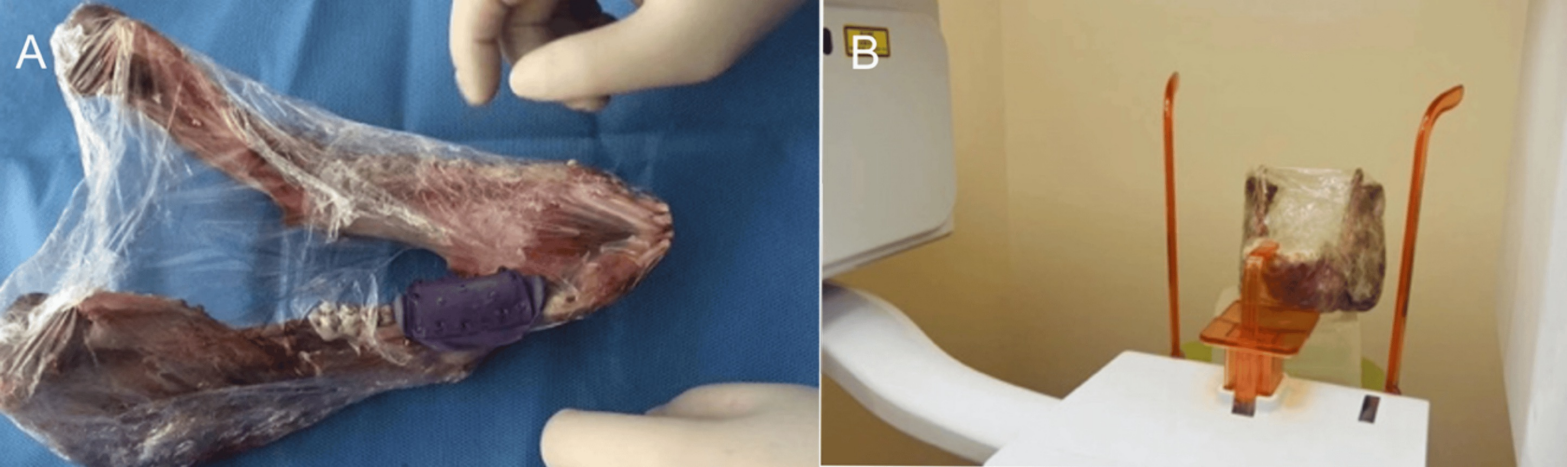
(A) Installation of the registration device. (B) CBCT imaging with the registration device installed CBCT: cone beam computed tomography

**Figure 2:**
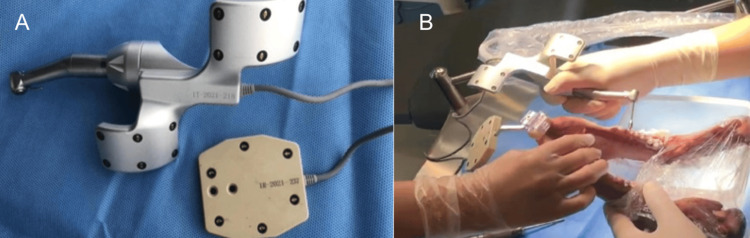
(A) Calibration and positioning of the surgical navigation equipment and mandibular locator. (B) Calibration and alignment of the surgical instruments and mandibular model

The registration device was installed on the porcine mandible, followed by CBCT imaging (Figures 1A, 1B). The data were imported into the navigation software for 3D reconstruction and non-invasive registration of landmarks. Based on the reconstructed 3D images, the proposed implantation positions were analyzed, and surgical instruments were prepared (Figure 2A, 2B).

During the surgery, the optical tracking device of the navigation system captured the positions of the drill tip and the porcine mandible. Calibration was performed, after which the mandibular model was matched to the virtual 3D reconstruction using registration points. After completing the calibration and registration process, the navigation system displayed the drill and surgical area in real time on the screen. Using an error control indicator, the navigation system guided the surgeon to perform the implantation procedure precisely as per the preoperative plan (Figure 2B).

**Figure 3:**
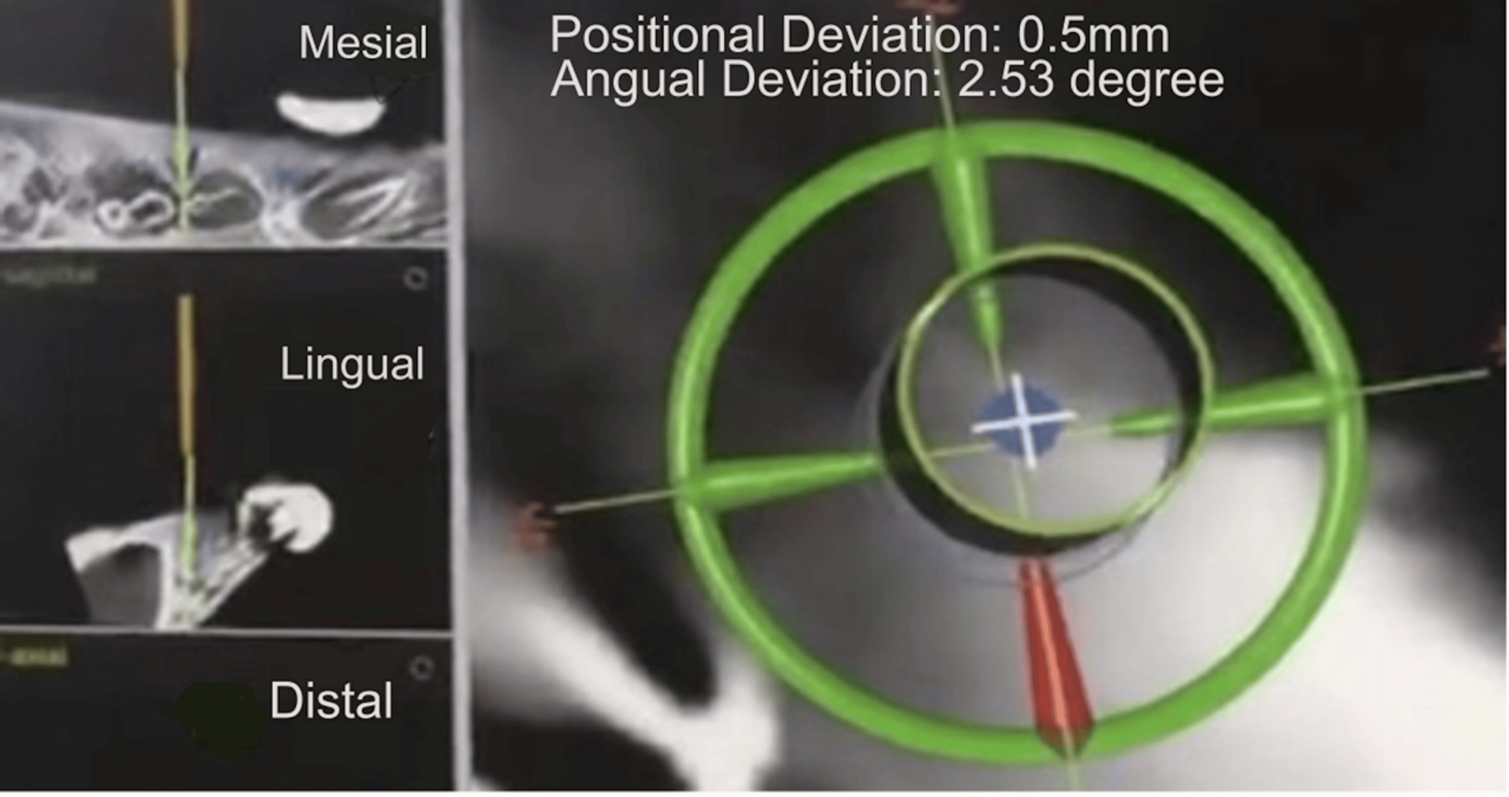
Real-time surgical navigation display during the implantation process

If deviations from the preoperative design occurred during the procedure, the surgeon could adjust the surgical plan in real time using the navigation software (Figure 3).

Post-Implantation Analysis With Dynamic Real-Time Navigation

**Figure 4:**
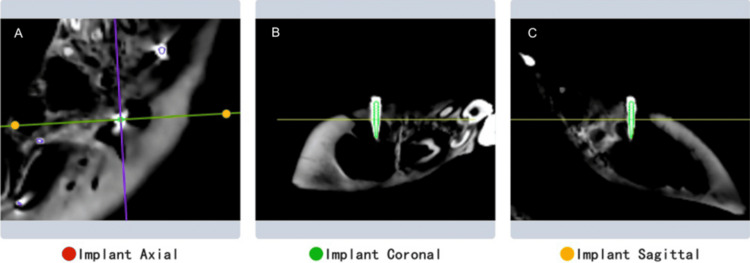
Overlay of the preoperative plan and postoperative CBCT image Visualization of the miniscrew position as planned pre-operatively (green outline) and its actual position post-operatively. Cross-sectional views showing the alignment of the planned and placed miniscrews. A: axial view; B: coronal view; and C: sagittal view CBCT: cone beam computed tomography

After implantation, CBCT scans were taken, and the 3D position data of the miniscrews were imported into the Dentsply Sirona implant accuracy verification software. The differences in entry points, endpoints, and angles between the actual and planned positions of the miniscrews were calculated by comparing pre-operative and post-operative data (Figure 4).

**Figure 5:**
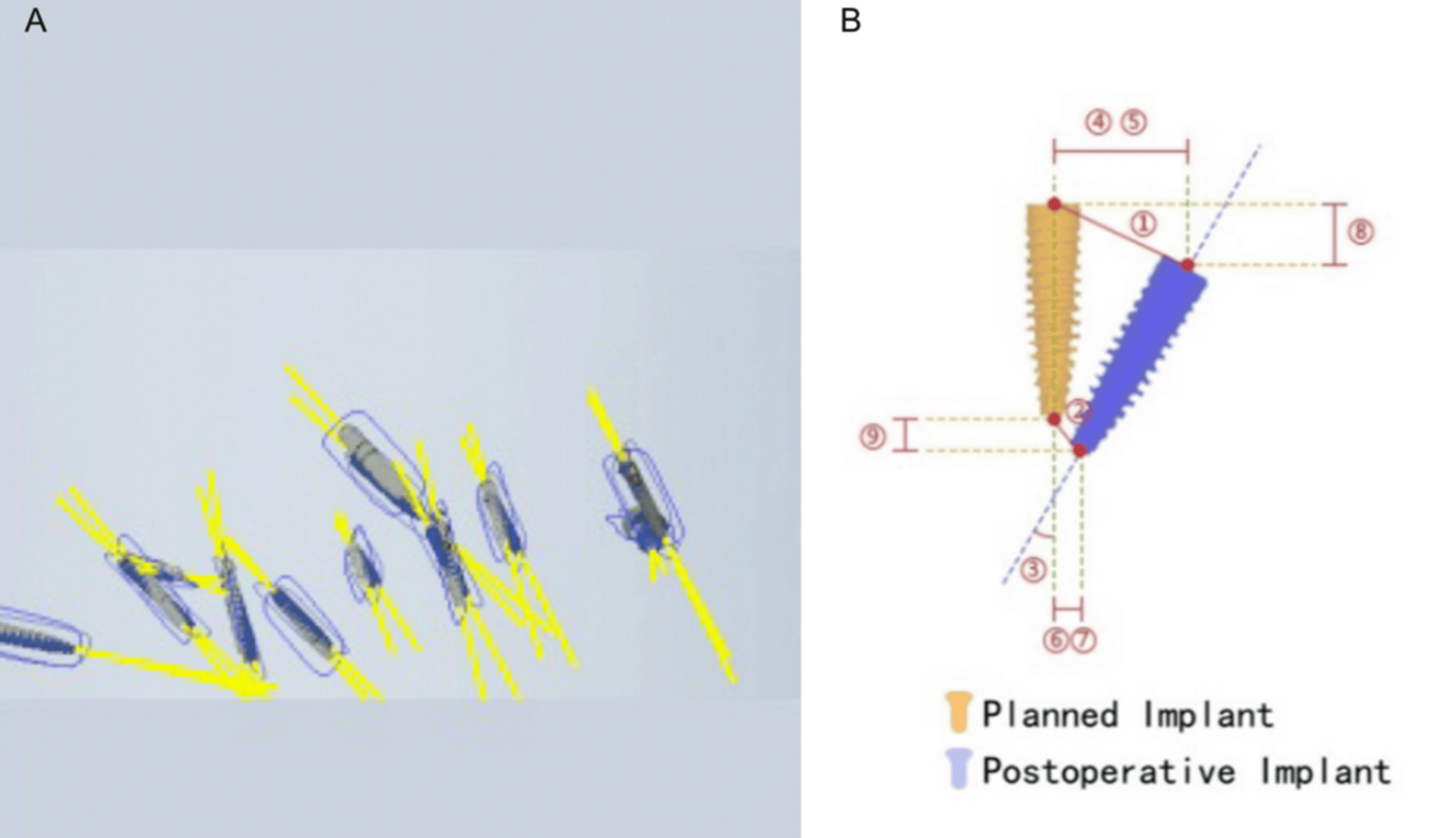
Visualization of miniscrew implantation accuracy A: Comparison between the planned miniscrew (blue) and actually placed miniscrew (grey) in a three-dimensional view; B: 1. Entry point deviation; 2. End point deviation; 3. Miniscrew angular deviation; 4. Buccolingual deviation at the entry point; 5. Mesiodistal deviation at the miniscrew entry point; 6. Buccolingual deviation at the miniscrew endpoint; 7. Mesiodistal deviation at the miniscrew endpoint; 8. Depth deviation at the miniscrew entry point; 9. Depth deviation at the miniscrew endpoint

A total of 30 orthodontic miniscrews were analyzed. The accuracy evaluation metrics included deviations in the entry points (buccolingual and mesiodistal directions), endpoints (buccolingual and mesiodistal directions), and angular deviations of the miniscrews (Figure 5).

Statistical analysis

All accuracy evaluation metrics were continuous variables. They were described and analyzed using mean, standard deviation, minimum and maximum values, median, and interquartile ranges. Box plots with scatter overlays were used for graphical representation. All statistical analyses and visualizations were performed using SAS 9.4 (SAS Institute Inc., Cary, NC).

## Results

**Figure 6:**
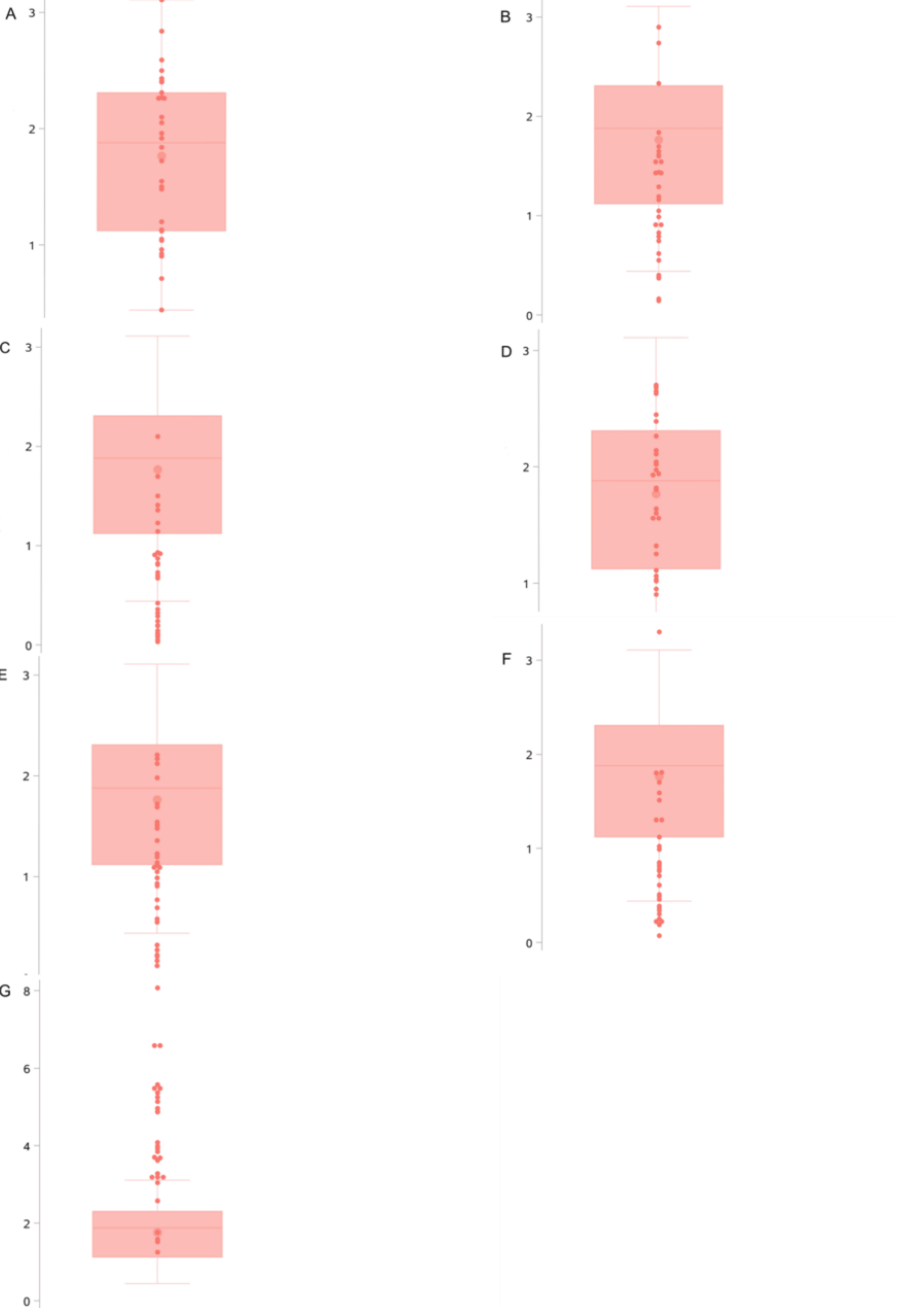
Deviation at the entry point, endpoint, and angle deviation of orthodontic miniscrew implants Box plots with scatter overlays showing the distribution of entry and endpoint deviations in millimeters and angle deviations in degrees. A. Entry point deviation; B. Buccolingual deviation at the entry point; C. Mesiodistal deviation at the entry point; D. Endpoint deviation; E. Buccolingual deviation at the endpoint; F. Mesiodistal deviation at the endpoint; G. Angular deviation

The results indicate that the minimum deviation at the miniscrew entry point was 0.4 mm, the maximum was 3.1 mm (with an average of 1.8 ± 0.7 mm), and a median (interquartile range) of 1.9 (1.1-2.3) mm. The minimum buccolingual deviation at the miniscrew entry point was 0.1 mm, the maximum was 2.9 mm (with an average of 1.2 ± 0.7 mm), and the median (interquartile range) of 1.2 (0.8-1.6) mm. The minimum mesiodistal deviation at the miniscrew entry point was 0 mm, the maximum was 2.1 mm (with an average of 0.7 ± 0.6 mm), and a median (interquartile range) of 0.7 (0.2-0.9) mm. There is more deviation in the buccolingual dimension than the mesiodistal dimension at the miniscrew entry points. The minimum deviation at the miniscrew endpoint was 0.6 mm, the maximum was 2.7 mm (with an average of 1.8 ± 0.6 mm), and the median (interquartile range) of 1.8 (1.3-2.1) mm. The minimum buccolingual deviation at the miniscrew endpoint was 0.1 mm, the maximum was 2.2 mm (with an average of 1.1 ± 0.6 mm), and the median (interquartile range) of 1.1 (0.6-1.5) mm. The minimum mesiodistal deviation at the miniscrew endpoint was 0.1 mm, the maximum was 3.3 mm (with an average of 0.9 ± 0.7 mm), and the median (interquartile range) of 0.8 (0.4-1.3) mm. There is more deviation in the buccolingual dimension than the mesiodistal dimension at the miniscrew endpoints. The angular deviation of miniscrew placement ranged from a minimum of 1.3° to a maximum of 8.1° (with an average of 4.1° ± 1.6°), and a median (interquartile range) of 3.9° (3.2°-5.3°). Detailed metrics are summarized in Table 1. The distribution of accuracy evaluation metrics is shown in Figure 6.

**Table 1:** Statistical results of implantation accuracy SD: Standard deviation; IQR: Interquartile range

Metric	Mean ± SD	Min-Max	Median (IQR)
Entry point deviation (mm)	1.8 ± 0.7	0.4–3.1	1.9 (1.1–2.3)
Endpoint deviation (mm)	1.8 ± 0.6	0.6–2.7	1.8 (1.3–2.1)
Angular deviation (°)	4.1 ± 1.6	1.3–8.1	3.9 (3.2–5.3)
Buccolingual deviation at entry (mm)	1.2 ± 0.7	0.1–2.9	1.2 (0.8–1.6)
Mesiodistal deviation at entry (mm)	0.7 ± 0.6	0.0–2.1	0.7 (0.2–0.9)
Buccolingual deviation at endpoint (mm)	1.1 ± 0.6	0.1–2.2	1.1 (0.6–1.5)
Mesiodistal deviation at endpoint (mm)	0.9 ± 0.7	0.1–3.3	0.8 (0.4–1.3)

## Discussion

This study aimed to evaluate the accuracy of orthodontic miniscrew implantation using a dynamic navigation system combined with CBCT imaging. The results indicate that the deviations in miniscrew entry points and endpoints under real-time dynamic navigation were minimal, ensuring the safety of miniscrew implantation. However, the maximum deviations observed may still pose potential safety concerns for the procedure. From the CBCT imaging data, no contact between the miniscrews and critical anatomical structures was detected. The angular deviations of the miniscrews at the entry point and endpoint were relatively small, meeting clinical treatment requirements.

Orthodontic miniscrews have become increasingly popular in clinical practice due to their simplicity, convenience, and adaptability in various positions. However, their stability and accuracy are influenced by multiple factors, which limit their clinical application. Major influencing factors include the implantation site, angle, screw length, and procedural stability during implantation. Among these, the accuracy of miniscrew implantation is the primary determinant of success. An important reason for screw loosening and failure is inaccurate implantation angles, which can result in the screws being placed too close to or in contact with adjacent roots, causing loosening and detachment [10].

Zhou et al. [11] showed through finite element analysis that miniscrews placed at least 1 mm away from the periodontal membrane ensured stability. Asscherickx et al. [12] further demonstrated a 100% success rate when the distance between the miniscrew and adjacent root exceeded 1 mm, assuming that other factors were controlled. However, even experienced clinicians face challenges due to anatomical constraints, implantation positions, and limitations of surgical tools, which may lead to accidental contact with roots or other complications. In this study, dynamic navigation successfully prevented any contact between orthodontic miniscrews and important anatomical structures.

With advancements in medical imaging and digital technology, Kim et al. [13] were the first to design guides for miniscrew placement using CBCT in 2007. Their approach significantly improved accuracy but faced limitations, such as guide instability, inability to adjust angles intraoperatively, lack of visibility in the surgical field, difficulty in removing the guide during surgery, and missing water-cooling system [14-16]. In contrast, dynamic navigation systems used in the current study allow real-time visualization under conditions, such as water cooling, enabling continuous or paused implantation of orthodontic miniscrews as needed.

Since 2000, real-time dynamic navigation systems have been applied in dental implantology [17]. Studies have shown that dynamic navigation improves the precision of implant placement. Kaewsiri et al. [18] conducted a randomized controlled trial comparing static and dynamic navigation systems for implant placement, showing comparable accuracy for both approaches. Similarly, other researchers [19-21] found that dynamic navigation systems provide higher precision, particularly for experienced clinicians. Block et al. [22] conducted a prospective study of placing 714 implants in 478 patients showing that the average angular deviation using dynamic navigation was 2.97° ± 2.09°, compared to 6.50° ± 4.21° for freehand implant placement.

While dynamic navigation has been widely studied in implantology, its application in orthodontic miniscrew placement has been limited. Previous studies using computer-aided design and computer-aided modeling (CAD-CAM) surgical guides reported average angular deviations of 4.60° ± 2.54° [23]. This study demonstrated that the average angular deviation using dynamic navigation was 4.1° ± 1.6°, slightly lower than that achieved with digital surgical guides. These results suggest that dynamic navigation can serve as a new and promising approach for orthodontic miniscrew implantation.

This study provides an accurate and safe method for miniscrew placement, offering a potential for broader clinical application in orthodontics. However, there are limitations. First, porcine mandibles were used as experimental models, and their dense cortical bone and high torque during implantation may have caused positional deviations or incomplete screw depth, potentially affecting accuracy and stability. Future studies should include well-designed clinical trials to obtain data closer to clinical scenarios. Second, the relatively high standard deviation observed in some measurements suggests that the sample size of this study was relatively small. Future research could consider increasing the sample size to improve reliability. Third, the design of animal tissue studies eliminates potential errors caused by patient motion and artifacts. However, future human studies should account for these errors in their design.

## Conclusions

The orthodontic miniscrew system designed with chairside dynamic navigation combined with CBCT imaging can be effectively used for the implantation of orthodontic miniscrews. The placement positions and angles of the miniscrews are relatively accurate, providing a practical foundation for further clinical applications in orthodontics.
